# Congenic Strains Confirm the Pleiotropic Effect of Chromosome 4 QTL on Mouse Femoral Geometry and Biomechanical Performance

**DOI:** 10.1371/journal.pone.0148571

**Published:** 2016-02-05

**Authors:** Jasmin Kristianto, Suzanne J. Litscher, Michael G. Johnson, Forum Patel, Mital Patel, Jacqueline Fisher, Ryley K. Zastrow, Abigail B. Radcliff, Robert D. Blank

**Affiliations:** 1 Department of Medicine, School of Medicine and Public Health, University of Wisconsin-Madison, Madison, Wisconsin, United States of America; 2 Department of Medicine, Medical College of Wisconsin, Milwaukee, Wisconsin, United States of America; 3 William S. Middleton Memorial Veterans Hospital, Madison, Wisconsin, United States of America; 4 Milwaukee VA Medical Center, Milwaukee, Wisconsin, United States of America; Université de Lyon—Université Jean Monnet, FRANCE

## Abstract

A pleiotropic quantitative trait locus (QTL) for bone geometry and mechanical performance in mice was mapped to distal chromosome 4 via an intercross of recombinant congenic mice HcB-8 and HcB-23. To study the QTL in isolation, we have generated C3H.B10-(*rs6355453-rs13478087*) (C.B.4.3) and C3H.B10-(*rs6369860-D4Mit170)* (C.B.4.2) congenic strains that harbor ~20 Mb and ~3 Mb, respectively, of chromosome 4 overlapping segments from C57BL/10ScSnA (B10) within the locus on a C3H/DiSnA (C3H) background. Using 3-point bend testing and standard beam equations, we phenotyped these mice for femoral mid-diaphyseal geometry and biomechanical performance. We analyzed the results via 2-way ANOVA, using sex and genotype as factors. In the C.B.4.3 strain, we found that homozygous B10/B10 male mice had smaller cross sectional area (CSA) and reduced total displacement than homozygous C3H/C3H mice. Sex by genotype interaction was also observed for maximum load and stiffness for C3H/C3H and B10/B10 mice, respectively. In C.B.4.2 strain, we found that homozygous B10/B10 mice had lower total displacement, post-yield displacement (PYD), stiffness, yield load and maximum load than mice harboring C3H allele. Sex by genotype interaction was observed in B10/B10 mice for perimeter, outer minor axis (OMA) and CSA. There were no significant differences in tissue level mechanical performance, which suggest that the QTL acts primarily on circumferential bone size. These data confirm the prior QTL mapping data and support other work demonstrating the importance of chromosome 4 QTL on bone modeling and bone responses to mechanical loading.

## Introduction

Bone biomechanical performance encompasses a suite of functionally related complex traits [1–4]. Approximately 70% of the variation in bone properties can be attributed to genetics [5, 6]. Many quantitative trait loci (QTL) for bone phenotypes have been mapped in mice and humans [7–9]. Several research groups, including ours, have mapped QTLs for bone traits in mice to distal chromosome 4 [10–13].

Mouse strains C57BL/10ScSnA and C3H/DiSnA mice have different bone geometry and biomechanical performance [14]. The HcB recombinant congenic strains were constructed by backcrossing male C57BL/10ScSnA (B10) to female C3H/DiSnA (C3H) mice for 3 generations and then inbreeding random offspring pairs to fixation. Twenty seven such strains were generated, each containing on average ~12.5% of alleles from B10, and the remaining alleles from C3H [15, 16]. Because they are inbred, they are homozygous throughout the genome [16, 17]. Because any pair of HcB strains share ~3/4 of their alleles, phenotypic differences between them can be attributed to the reduced number of segregating loci. In early experiments, we found that HcB-23 mice had larger, stiffer and stronger bone than HcB-8 [14, 18]. We therefore sought to identify the genomic regions mediating those differences by performing an intercross to map quantitative trait loci (QTLs) for bone biomechanical performance in these strains. We found a pleiotropic chromosome 4 QTL affecting bone shape, size and strength [10, 19–21].

To study the QTL in isolation from other segregating loci, we have generated two congenic strains that carry overlapping segments of chromosome 4 from C57BL/10ScSnA (B10) within the locus on a C3H/DiSnA (C3H) background. The long C3H.B10-(*rs6355453-rs13478087*) (hereafter abbreviated as C.B.4.3) and short C3H.B10-(*rs6369860-D4Mit170)* (hereafter abbreviated as C.B.4.2) congenic strains harbor a ~20 Mb and a ~3 Mb B10-derived chromosome segment, respectively. We report biomechanical properties of F2 mice obtained by intercrossing the congenic strains to the recipient parent, C3H. The mice studied here are complementary to other congenic strains covering this region, in that our mice examine introgression of B10 alleles onto a C3H background, rather than introgression of C3H alleles onto a C57BL/6J background. Our study sought to validate the linkage findings from the intercross and confirm the pleiotropic effect of chromosome 4 QTL on a different donor/background combination. We hypothesize that chromosome 4 QTL affects bone geometry and biomechanical performance in both congenic strains.

Sex differences in bone geometry and biomechanical performance is well documented [22–26]. Male mice develop larger and stronger bone than female mice [22, 23]. Clinical studies showed that males have larger skeletal size and bone mass than females [27–29]. Females are also at higher risk for fractures than males [30, 31]. There are many instances in which allelic differences affect males and females differently [32–34]. Thus, we also examined sex by genotype interaction in both congenic strains.

## Materials and Methods

### Mice

The parental mice in this study were the recombinant congenic strains HcB-8, HcB-13, and the recipient progenitor of those strains, C3H/DiSnA. The congenic strains were constructed by backcrossing the donor strains (HcB-8 and HcB-13) to the recipient strain C3H/DiSnA to yield the long congenic strain C.B.4.3 and the short congenic strain C.B.4.2, respectively. Markers used to genotype the congenic segment are listed in Table 1. Using marker assisted selection, the time frame for the congenic strain derivation was 5 backcrosses [35]. At the completion of backcrossing, males and females heterozygous for the congenic segment were intercrossed to yield the congenic strains. Male congenic mice were intercrossed with C3H/DiSnA female mice to yield a F2 cohort for phenotyping. We maintained the animals to an age of 17 ± 1 weeks, because this is the age at which mice achieve peak bone mass [36]. Mice were housed 2–5 mice/500 cm^2^ cage with 12 h light-dark cycling, given autoclaved tap water, and fed laboratory rodent chow 5001 (PMI Nutrition International, Richmond, IN, USA) *ad libitum*. Animals were euthanized by CO_2_ inhalation, following American Veterinary Medical Association recommendations. Immediately following euthanasia, animals were weighed and measured (rostro-anal length), and femora and humeri were dissected free of soft tissue for additional phenotyping. Bones were wrapped in PBS-saturated gauze and stored frozen at −80°C. The animal protocol was approved by the University of Wisconsin and the William S. Middleton Memorial Veterans Hospital institutional animal care and use committees.

**Table 1:** Markers for C.B.4.3 and C.B.4.2.

Map Location (cM)	Genome Coordinates (bp)	Markers	C.B.4.3	C.B.4.2
64.5	131080896	rs6355453	**B10**	C3H
66.25	133427367–133427471	D4Mit204	C3H	C3H
68	135736871–135737020	D4Mit134	C3H	C3H
68	135872315	rs13478000	**B10**	C3H
68.55	136361074–136361203	D4Mit69	C3H	C3H
69	136674649	rs13478002	**B10**	C3H
70	137147030	***rs6369860***	**B10**	**B10**
70.02	137890537–137890684	***D4Mit54***	**B10**	**B10**
70.47	138615338–138615442	***D4Mit170***	**B10**	**B10**
74.75	141261981	D4Mit32	C3H	C3H
78.17	145057656–145057773	D4Mit232	C3H	C3H
78.1	144973088	rs4224923	**B10**	C3H
81.4	150309421	rs13478048	**B10**	C3H
81.52	150935515	rs13478050	**B10**	C3H
83.63	153217171	rs13478062	**B10**	C3H
84.7	154430058	rs13478089	**B10**	C3H
87.6	155234755	rs13478071	**B10**	C3H
88.4	155507226	rs13478087	**B10**	C3H

For the long chromosome 4 congenic strain (C.B.4.3) mice, we studied a total of 52 mice. We performed mechanical testing on 9 C3H/C3H males, 8 C3H/C3H females, 8 C3H/B10 males, 9 C3H/B10 females, 10 B10/B10 males and 8 B10/B10 females. For the short chromosome 4 congenic strain (C.B.4.2), we studied a total of 54 mice. We performed mechanical testing on 10 C3H/C3H males, 4 C3H/C3H females, 17 C3H/B10 males, 8 C3H/B10 females, 9 B10/B10 males and 6 B10/B10 females. The differences in sample sizes were due to chromosomal segregation.

### Genotyping

We genotyped the F2 progenies at microsatellite and SNP markers (Table 1) using standard methods [10]. Genetic and physical locations of markers and genes are from the Mouse Genome Database, MGI 6.0 (http://www.informatics.jax.org/genes.shtml). Only non-recombinant mice were held for phenotyping.

### Phenotyping

#### Bone Mineral Density (BMD)

We measured areal BMD of isolated femora by dual X-ray absorptometry (DXA) as described previously [37]. Briefly, each bone was scanned twice with repositioning. Measured BMD was adjusted for position on the scanning grid, and the average of the duplicate measurements was used as that bone’s BMD. We averaged both femora to obtain the animal’s BMD. We measured femoral length between the greater trochanter and the medial condyle with Vernier calipers, using the average value of both femora from each animal.

#### Three-point Bending Test

We tested femoral diaphysis biomechanical performance by quasi-static 3-point bending under displacement control at a rate of 0.3 mm/s, with a support span of 7.5 mm following the same methods as in prior work [10]. Before mechanical testing, bones were gradually warmed to room temperature and hydrated in PBS. We tested both femora from each animal and used the average value. All bones were subjected to 2 freeze-thaw cycles before testing. Femora were supported at the condyles and the third trochanter, with the condyles oriented downward, producing a mid-diaphyseal fracture. Following fracture, we obtained digital photographs of the fracture plane of each bone and analyzed the images with SigmaScan Pro 5.1 (SPSS, Chicago, IL, USA), extracting the periosteal perimeter, cortical cross-sectional area (CSA), outer and inner major and minor axis lengths, shape factor (ratio of outer major axis length to outer minor axis length), cross-sectional moment of inertia (a measure of the distribution of material around a neutral axis), and slenderness (ratio of femoral length to mid diaphyseal perimeter) [10]. Load-displacement curves were analyzed to determine total and post-yield displacement, yield load, maximum load, stiffness, and energy to failure. The whole-bone mechanical properties and the dimensions of the cross-section obtained from the digital photographs were used to calculate tissue-level mechanical properties according to standard beam equations [20].

### Statistical Analyses

Comparisons of intercross subgroups were by 2-way ANOVA, with genotype and sex as factors, with *post hoc* evaluation of significant differences between groups by the Holm-Sidak test. The raw data for majority of the analyzed traits satisfied the parametric assumptions for ANOVA, with the exception of slenderness. Thus, we transformed the raw data for slenderness to satisfy the assumptions for ANOVA. Statistical analyses were performed with SigmaStat 3.5 (SPSS). Summary data in all figures and tables are shown as means ± SE.

## Results

### Bone Geometry and Biomechanical Performance of C.B.4.3 Mice

We studied a total of 52 chromosome 4 long congenic segment (C3H x C.B.4.3) F2 mice including 9 C3H/C3H males, 8 C3H/C3H females, 8 C3H/B10 males, 9 C3H/B10 females, 10 B10/B10 males and 8 B10/B10 females.

**Fig 1:**
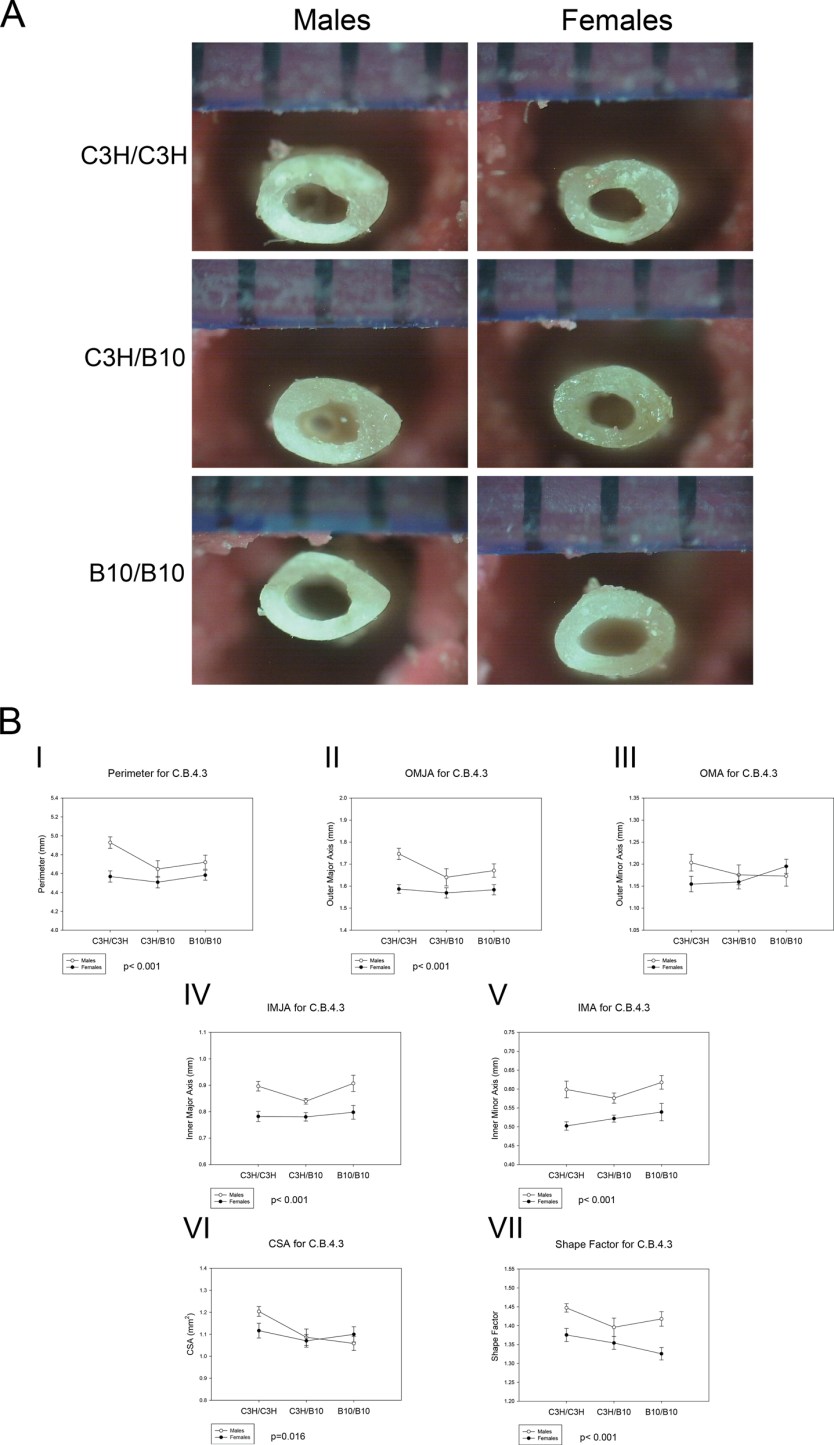
Femoral Geometry of C.B.4.3 Mice. (A) The representative photographs of C.B. 4.3 femur cross-sections following 3-point bending test for each genotype. (B) The effects of genotype on (I) Perimeter, p < 0.001, sex differences across all genotypes; (II) OMJA, p < 0.001 sex differences across all genotypes; (III) OMA, no significant differences; (IV) IMJA, p < 0.001, sex differences across all genotypes; (V) IMA, p < 0.001, sex differences across all genotypes; (VI) CSA, p = 0.016, B10/B10 and C3H/B10 < C3H/C3H for both sexes; (VII) Shape factor, p < 0.001, sex differences across all genotypes and genotype differences between C3H/C3H with both C3H/B10 and B10/B10. Values are means ± SE.

There were no significant differences in body weight, body length and femoral length between the genotypes (Table A in S1 File). There was no significant difference in BMD between the genotypes. Representative photographs of the femora following the 3-point bending test are shown in Fig 1(A).

#### Femoral Geometry of C.B.4.3 Mice

The bone geometry measurements are summarized in Table 2 and Fig 1(B). Outer major axis (OMJA), cross sectional area (CSA), cross sectional moment of inertia (MOI), shape factor and slenderness differed by genotypes regardless of sex. The OMJA of C3H/B10 mice was smaller than C3H/C3H mice (p = 0.037). However, there was no significant difference in OMJA between C3H/C3H and B10/B10 mice. Regardless of sex, both C3H/B10 and B10/B10 mice also had smaller femoral cross sectional area (CSA) than C3H/C3H mice (p = 0.016). Both C3H/B10 and B10/B10 mice had more circular cross sections than C3H/C3H mice (p = 0.023). Cross sectional moment of inertia (MOI) of B10/B10 mice were larger than C3H/B10 mice MOI (p = 0.042), but not significantly different from C3H/C3H mice. Both C3H/C3H and B10/B10 mice had more slender bones than C3H/B10. Perimeter, OMJA, inner major axis (IMJA), inner minor axis (IMA), and shape factor differed by sex. Females had smaller perimeter (p<0.001), OMJA (p<0.001), IMJA (p<0.001) and IMA (p<0.001) than males across all genotypes. Females had more circular cross sections than males (p<0.001). Across all genotypes, male mice had more slender bone than female mice (p = 0.004). We also observed sex by genotype interactions in C3H/C3H (p = 0.001) and B10/B10 (p = 0.010) mice for slenderness. There were no significant differences in outer minor axis (OMA) across all sex and genotypes. Based on these data, the C.B.4.3 congenic segment appears to regulate differences in femoral cross sectional geometry, with mice harboring B10 allele have smaller bones than homozygous C3H mice.

**Table 2:** Femoral Geometry of C.B.4.3 Mice.

C.B. 4.3						
	MALES			FEMALES		
	C3H/C3H (9)	C3H/B10 (8)	B10/B10 (10)	C3H/C3H (8)	C3H/B10 (9)	B10/B10 (8)
**Perimeter (mm)**^**B**^	4.93 ± 0.06	4.64 ± 0.08	4.72 ± 0.07	4.57 ± 0.06	4.51 ± 0.06	4.58 ± 0.05
**Outer major axis (mm)** ^**A**^^,^^**B**^	1.76 ± 0.01	1.64 ± 0.03	1.67 ± 0.03	1.58 ± 0.04	1.57 ± 0.02	1.58 ± 0.02
**Outer minor axis (mm)**	1.20 ± 0.01	1.17 ± 0.02	1.17 ± 0.02	1.15 ± 0.02	1.16 ± 0.02	1.19 ± 0.02
**inner major axis (mm)**^**B**^	0.90 ± 0.02	0.83 ± 0.01	0.91 ± 0.03	0.78 ± 0.02	0.78 ± 0.02	0.80 ± 0.03
**inner minor axis (mm)**^**B**^	0.59 ± 0.07	0.57 ± 0.01	0.62 ± 0.02	0.50 ± 0.01	0.52 ± 0.01	0.54 ± 0.02
**CSA (mm**^**2**^**)**^**A**^	1.20 ± 0.02	1.09 ± 0.03	1.06 ± 0.03	1.12 ± 0.03	1.07 ± 0.03	1.09 ± 0.03
**MOI (mm**^**4**^**)**^**A**^	0.13 ± 0.03	0.10 ± 0.003	0.14 ± 0.02	0.12 ± 0.01	0.12 ± 0.01	0.15 ± 0.01
**Shape factor (unitless)**^**A**^^,^^**B**^	1.44 ± 0.01	1.40 ± 0.02	1.40 ± 0.02	1.38 ± 0.02	1.35 ± 0.02	1.33 ± 0.02
**Slenderness (unitless)**^**A**^^,^^**C**^	3.01 ± 0.05	3.20 ± 0.06	3.06 ± 0.06	3.26 ± 0.04	3.29 ± 0.04	3.31 ± 0.05

Values are means ± SE; N, numbers of mice. Data analyzed by 2 way ANOVA

^A^ genotype effect

^B^ sex effect

^C^ sex by genotype interaction

#### Femoral Mechanical Properties of C.B.4.3 Mice

**Fig 2:**
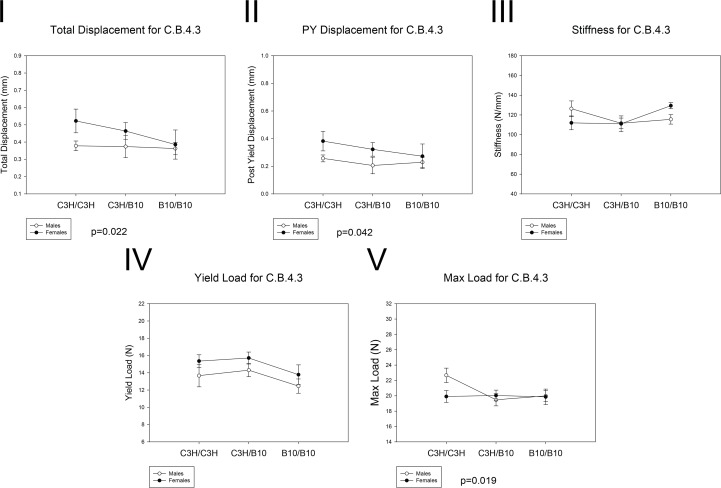
Whole Bone Mechanical Performance of C.B.4.3 mice. Open circle: males. Black circle: females. The effects of genotype on (I) total displacement, B10/B10 < C3H/C3H (p = 0.022). (II) PYD, p = 0.042 for sex differences across all genotypes. (V) Max load, p = 0.019 for differences in males (B10/B10 and C3H/B10 < C3H/C3H). No significant differences in (III) stiffness and (IV) yield load between genotypes.

The whole-bone biomechanical performance is summarized in Table 3 and Fig 2. Total displacement, or total amount of femur deformation before fracture, was significantly lower in B10/B10 mice compared to C3H/C3H mice (p = 0.022). C3H/C3H male mice have higher maximum (max) load than C3H/B10 and B10/B10 male mice (p = 0.019), but the differences were not significant in females. However, C3H/C3H and C3H/B10 females had greater total displacement than C3H/C3H, C3H/B10 and B10/B10 males (p<0.001). The post yield displacement (PYD), a measure of ductility, was significantly lower in males than females across all genotypes (p = 0.042). There were no significant differences in yield load, stiffness and energy between genotypes. Sex by genotype interaction was observed for max load. Male C3H/C3H mice had higher max loads than any other groups; the other groups did not differ from each other (p = 0.037). Sex by genotype interaction was also observed for stiffness, in which C3H/C3H males had greater stiffness than males of other genotypes, while B10/B10 females had greater stiffness than females of other genotypes (p = 0.036). Generally, mice harboring C3H allele had greater phenotypic value than homozygous B10/B10 mice. Thus, these results showed that male mice harboring B10 allele in their chromosome 4 congenic segment had reduced total displacement and had lower max load than homozygous C3H/C3H mice.

**Table 3:** Whole-Bone Mechanical Performance of C.B.4.3 Mice.

C.B. 4.3						
	MALES			FEMALES		
	C3H/C3H (9)	C3H/B10 (8)	B10/B10 (10)	C3H/C3H (8)	C3H/B10 (9)	B10/B10 (8)
**total displacement (mm)** ^**A**^	0.38 ± 0.03	0.34 ± 0.06	0.36 ± 0.04	0.52 ± 0.07	0.46 ± 0.05	0.39 ± 0.08
**post-yield displacement (mm)**^**B**^	0.26 ± 0.03	0.21 ± 0.06	0.23 ± 0.04	0.38 ± 0.07	0.32 ± 0.05	0.28 ± 0.09
**stiffness (N/mm)**^**C**^	126 ± 8	112 ± 5	115 ± 5	112 ± 7	111 ± 8	129 ± 3
**yield Load (N)**	13.7 ± 1.3	14.3 ± 0.7	12.6 ± 1.0	15.4 ± 0.7	15.7 ± 0.7	13.8 ± 1.1
**max load (N)**^**A**^^,^^**C**^	22.7 ± 0.9	19.5 ± 0.8	20.0 ± 0.8	19.9 ± 0.8	20.0 ± 0.7	20.0 ± 1.0
energy (N*mm)	5.54 ± 0.45	4.28 ± 0.57	4.48 ± 0.30	4.12 ± 0.34	4.41 ± 0.43	4.51 ± 0.56

Values are means ± SE; N, numbers of mice. Data analyzed by 2 way ANOVA

^A^ genotype effect

^B^ sex effect

^C^ sex by genotype interaction

#### Tissue Level Properties of C.B.4.3 Mice

Tissue level biomechanical performance is summarized in Table B in S1 File. Males had greater post yield strain (p = 0.010) than females across all genotypes. However, there were no significant differences in other tissue level properties across all genotypes.

#### Additive and Dominance Effects of C.B.4.3 Mice

Genotype-dependent differences in trait values can be partitioned into additive and dominance effects. These are defined and summarized in Table 4. For OMJA and MOI, the locus had a significant additive effect in both sexes. For CSA, the locus had a significant dominance effect in males and a significant additive effect in females. The locus had a significant dominance effect in both sexes for shape factor. In males C.B.4.3, the locus had a larger dominance effect for slenderness that lead to the heterozygous having thicker femora than either homozygote. The locus had a larger additive effect for slenderness in females. For total displacement, the locus had a significant additive effect in males and a larger dominance effect in females. We averaged males and females data for each genotype to compare the additive versus dominance effect in a whole population. The data showed the locus having a significant dominant effect on slenderness and total displacement, while larger additive effect was measured for OMJA, MOI and max load. In summary, these findings suggested that the mechanical effect of the QTL could be attributed primarily to differences in bone size.

**Table 4:** Additive and Dominance Effects for Chromosome 4 QTL in C.B.4.3 Mice.

C.B. 4.3					
	**MALES**				
** **	**C3H/C3H (9)**	**C3H/B10 (8)**	**B10/B10 (10)**	**additive effect**	**dominance effect**
**Outer major axis (mm)** ^**A**^^,^^**B**^	1.76 ± 0.01	1.64 ± 0.03	1.67 ± 0.03	-0.05	-0.08
**CSA (mm**^**2**^**)**^**A**^	1.20 ± 0.02	1.09 ± 0.03	1.06 ± 0.03	-0.07	-0.04
**MOI (mm**^**4**^**)**^**A**^	0.13 ± 0.03	0.10 ± 0.003	0.14 ± 0.02	0.01	-0.04
**Shape factor (unitless)**^**A**^^,^^**B**^	1.44 ± 0.01	1.40 ± 0.02	1.40 ± 0.02	-0.02	-0.02
**Slenderness (mm/mm)**^**A**^^,^^**C**^	3.01 ± 0.05	3.20 ± 0.06	3.06 ± 0.06	0.03	0.17
**total displacement (mm)** ^**A**^	0.38 ± 0.03	0.34 ± 0.06	0.36 ± 0.04	-0.01	-0.03
**max load (N)**^**A**^^,^^**C**^	22.7 ± 0.9	19.5 ± 0.8	20.0 ± 0.8	-1.35	-1.85
	**FEMALES**				
** **	**C3H/C3H (8)**	**C3H/B10 (9)**	**B10/B10 (8)**	**additive effect**	**dominance effect**
**Outer major axis (mm)** ^**A**^^,^^**B**^	1.58 ± 0.04	1.57 ± 0.02	1.58 ± 0.02	0	-0.01
**CSA (mm**^**2**^**)**^**A**^	1.12 ± 0.03	1.07 ± 0.03	1.09 ± 0.03	-0.015	-0.035
**MOI (mm**^**4**^**)**^**A**^	0.12 ± 0.01	0.12 ± 0.01	0.15 ± 0.01	0.015	-0.015
**Shape factor (unitless)**^**A**^^,^^**B**^	1.38 ± 0.02	1.35 ± 0.02	1.33 ± 0.02	-0.025	-0.005
**Slenderness (mm/mm)**^**A**^^,^^**C**^	3.26 ± 0.04	3.29 ± 0.04	3.31 ± 0.05	0.025	0.005
**total displacement (mm)** ^**A**^	0.52 ± 0.07	0.46 ± 0.05	0.39 ± 0.08	-0.065	0.005
**max load (N)**^**A**^^,^^**C**^	19.9 ± 0.8	20.0 ± 0.7	20.0 ± 1.0	0.05	0.05
	**MALES AND FEMALES AVERAGE **				
	**C3H/C3H (17)**	**C3H/B10 (17)**	**B10/B10 (18)**	**additive effect**	**dominance effect**
**Outer major axis (mm)** ^**A**^^,^^**B**^	1.67	1.61	1.63	-0.02	-0.04
**CSA (mm**^**2**^**)**^**A**^	1.16	1.08	1.08	-0.04	-0.04
**MOI (mm**^**4**^**)**^**A**^	0.125	0.11	0.15	0.01	-0.03
**Shape factor (unitless)**^**A**^^,^^**B**^	1.41	1.38	1.37	-0.02	-0.01
**Slenderness (mm/mm)**^**A**^^,^^**C**^	3.135	3.25	3.19	0.03	0.09
**total displacement (mm)** ^**A**^	0.45	0.40	0.38	-0.04	-0.01
**max load (N)**^**A**^^,^^**C**^	21.3	19.75	20.00	-0.65	-0.90

Values are means ± SE; N, numbers of mice.

^A^ genotype effect

^B^ sex effect

^C^ sex by genotype interaction

Additive effect = ½ [average phenotype (C3H/C3H)–average phenotype (B10/B10)]

Dominance effect = average phenotype (C3H/B10)– ½ [average phenotype (C3H/C3H) + average phenotype (B10/B10)]

### Bone Geometry and Biomechanical Performance of C.B.4.2 Mice

We studied a total of 54 short chromosome 4 congenic segment (C3H x C.B.4.2) F2 mice, including 10 C3H/C3H males, 4 C3H/C3H females, 17 C3H/B10 males, 8 C3H/B10 females, 9 B10/B10 males and 6 B10/B10 females.

**Fig 3:**
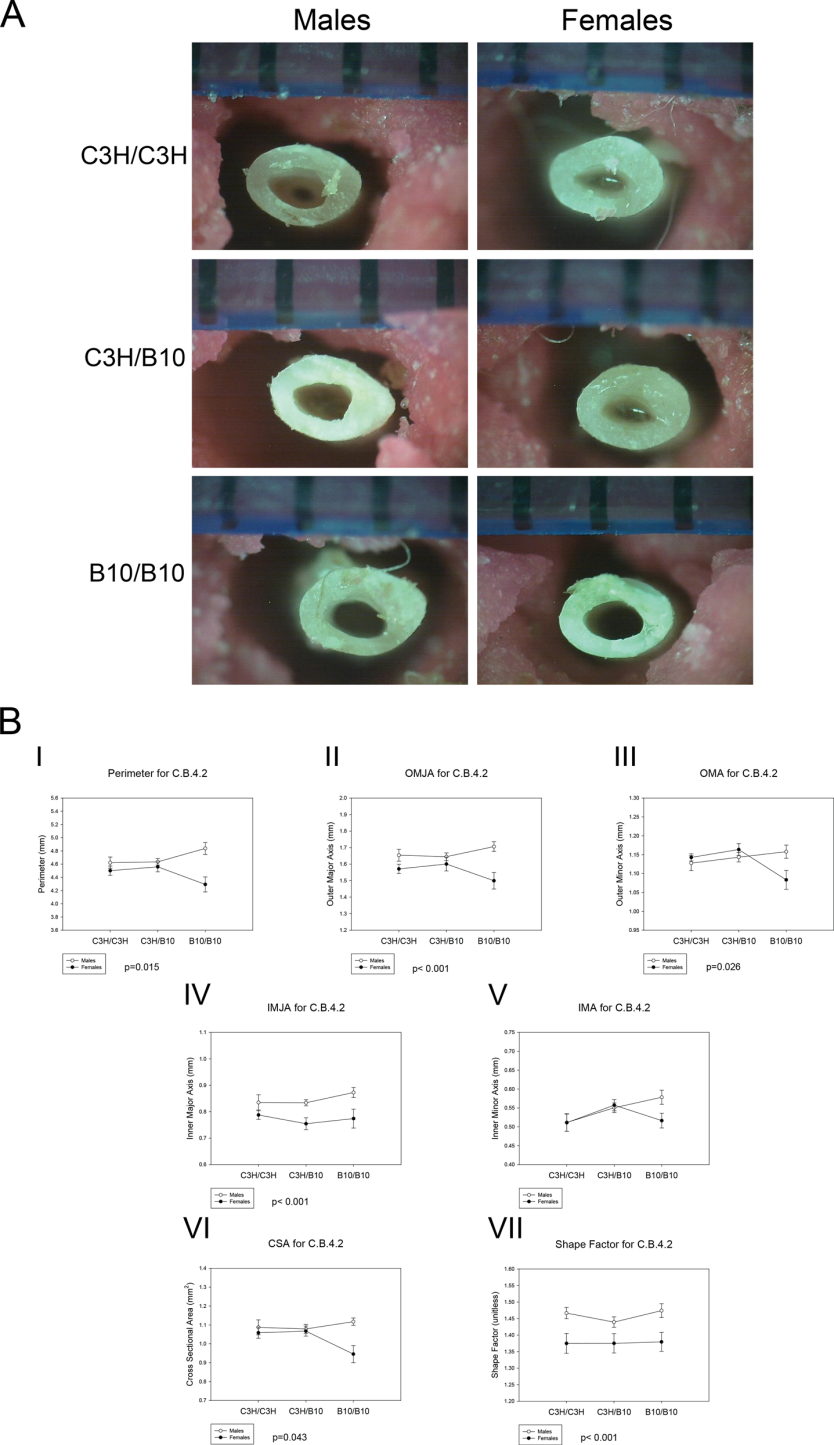
Femoral Geometry of C.B.4.2 mice. (A) Representative photographs of C.B.4.2 femur cross sections following 3-point bending test for each genotype. (B) The effects of sex and genotype on (I) perimeter, p = 0.015, sex by genotype interactions B10/B10 females < B10/B10 males; (II) OMJA, p < 0.001, sex differences across all genotypes, (III) OMA, p = 0.026, sex by genotype interactions B10/B10 females < B10/B10 males and genotype differences in B10/B10 females < C3H/B10 females, (IV) IMJA, p < 0.001, sex differences across all genotypes, and (V) IMA, no significant differences; (VI) CSA, p = 0.043, sex by genotype interaction B10/B10 females < B10/B10 males and genotype differences in B10/B10 females < C3H/B10 and C3H/C3H females (p = 0.045); (VII) Shape factor, p < 0.001, sex differences across all genotypes. Values are means ± SE.

There were no significant differences in body weight, body length, or femoral length between genotypes (Table C in S1 File). There was also no significant difference in BMD between the genotypes. Representative pictures of the femora following the three-point bending test are shown in Fig 3(A).

#### Femoral Geometry of C.B.4.2 Mice

Femoral mid-diaphysis geometric analysis following 3-point bending test are summarized in Table 5 and Fig 3 (B). B10/B10 females had significantly smaller CSA (p = 0.045) than C3H/C3H and C3H/B10 females and smaller OMA than C3H/B10 females (p = 0.016). On average, B10/B10 males tended toward higher CSA relative to other genotypes, but this did not reach statistical significance. There were no significant differences in OMA between the males. Across all genotypes, males had greater OMJA (p<0.001) and IMJA (p<0.001) than females. However, there were no significant differences in IMA and MOI between sex and genotypes. Females had more circular cross sections than males (p<0.001) across all genotypes. Across all genotypes, male mice had more slender bones than females (p<0.001). Sex by genotype interaction was evident in homozygous B10/B10 mice. B10/B10 females had smaller perimeter (p = 0.015), OMA (p = 0.026) and CSA (p = 0.043) than B10/B10 males. Based on the femoral mid-diaphysis structure analysis, the short congenic segment of C.B.4.2 appeared to modulate differences between males and females femora geometry, in particular in homozygous B10/B10 mice. The effect of genotypes was primarily observed in the female mice. These data demonstrated that the short congenic segment of chromosome 4 in C.B.4.2 regulates size and shape differences between sexes and between females of different genotypes.

**Table 5:** Femoral Geometry of C.B.4.2 Mice.

C.B. 4.2						
	MALES			FEMALES		
	C3H/C3H (10)	C3H/B10 (17)	B10/B10 (9)	C3H/C3H (4)	C3H/B10 (8)	B10/B10 (6)
**Perimeter (mm)**^**C**^	4.62 ± 0.08	4.63 ± 0.05	4.85 ± 0.09	4.50 ± 0.07	4.56 ± 0.08	4.29 ± 0.11
**Outer major axis (mm)**^**B**^	1.65 ± 0.03	1.65 ± 0.02	1.71 ± 0.03	1.57 ± 0.02	1.60 ± 0.04	1.50 ± 0.05
**Outer minor axis (mm)**^**A**^^,^^**C**^	1.13 ± 0.02	1.14 ± 0.01	1.16 ± 0.02	1.14 ± 0.01	1.16 ± 0.02	1.08 ± 0.03
**inner major axis (mm)**^**B**^	0.84 ± 0.03	0.83 ± 0.01	0.88 ± 0.02	0.79 ± 0.02	0.75 ± 0.02	0.77 ± 0.04
**inner minor axis (mm)**	0.51 ± 0.02	0.55 ± 0.01	0.57 ± 0.04	0.51 ± 0.02	0.52 ± 0.01	0.52 ± 0.02
**CSA (mm**^**2**^**)**^**A**^^,^^**C**^	1.08 ± 0.04	1.08 ± 0.02	1.14 ± 0.02	1.05 ± 0.03	1.07 ± 0.03	0.95 ± 0.05
**MOI (mm**^**4**^**)**	0.14 ± 0.02	0.14 ± 0.01	0.12 ± 0.01	0.14 ± 0.01	0.14 ± 0.02	0.13 ± 0.02
**Shape factor (unitless)**^**B**^	1.47± 0.02	1.44 ± 0.02	1.47 ± 0.02	1.38 ± 0.03	1.38 ± 0.03	1.38 ± 0.03
**Slenderness (unitless)**^**B**^	3.01 ± 0.06	3.10 ± 0.03	3.04 ± 0.05	3.20 ± 0.07	3.20 ± 0.06	3.20 ± 0.06

Values are means ± SE; N, numbers of mice. Data analyzed by 2 way ANOVA

^A^ genotype effect

^B^ sex effect

^C^ sex by genotype interaction

#### Femoral Mechanical Properties of C.B.4.2 Mice

**Fig 4:**
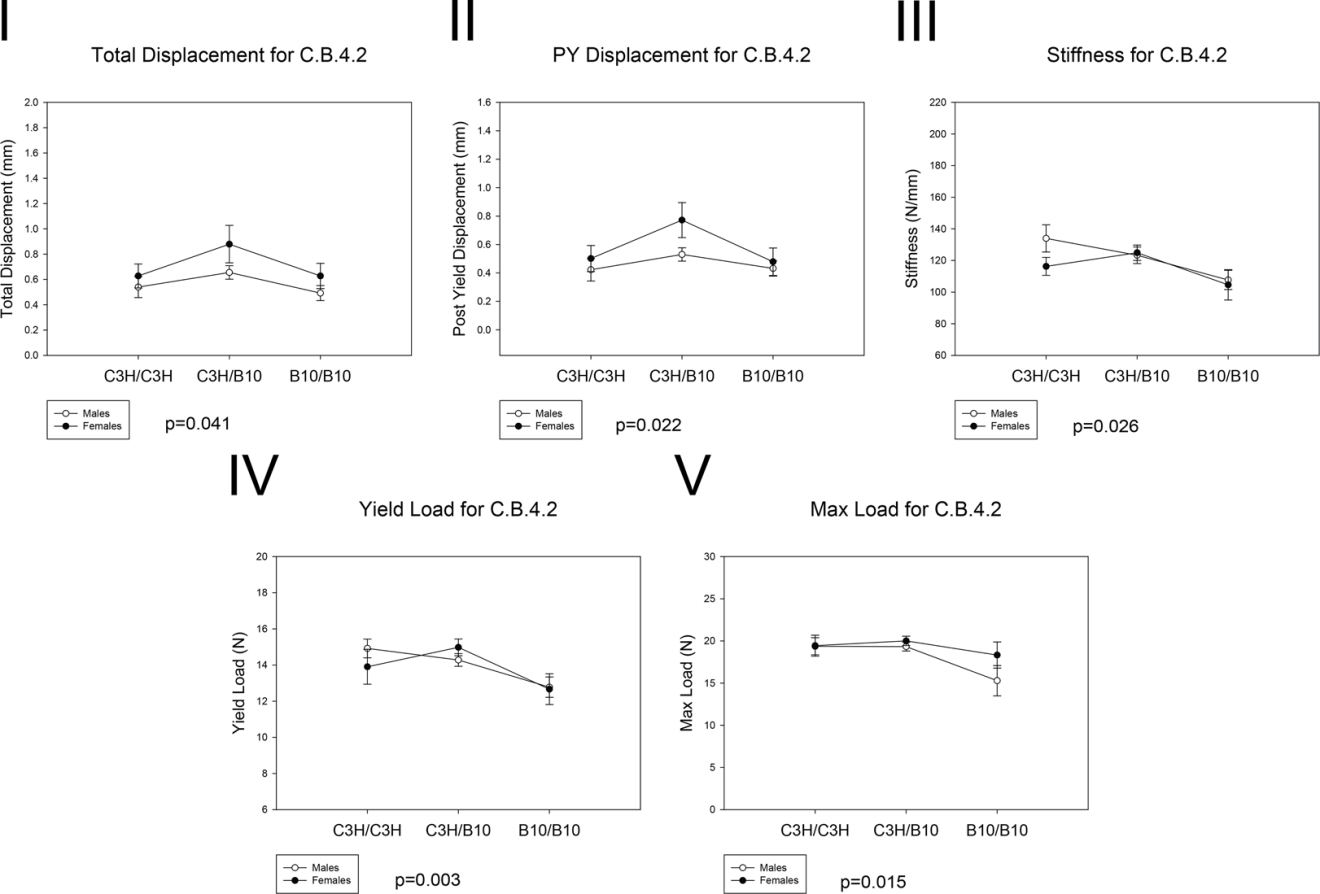
Whole Bone Mechanical Performance of C.B.4.2 mice. The effects of sex and genotypes on (I) Total displacement, p = 0.041, B10/B10 < C3H/B10 for both sexes; (II) PYD, p = 0.022, B10/B10 < C3H/B10 for both sexes, (III) Stiffness, p = 0.026, B10/B10 < C3H/B10 for both sexes; (IV) Yield load, p = 0.003, B10/B10 < C3H/B10 and C3H/C3H for both sexes, (V) Max load, p = 0.015, B10/B10 < C3H/B10 and C3H/C3H for both sexes. Values are means ± SE

The whole-bone biomechanical performance is summarized in Table 6 and Fig 4. B10/B10 mice had lower total displacement (p = 0.041), PYD (p = 0.022) and stiffness (p = 0.026) than C3H/B10 mice. Furthermore, B10/B10 femora had lower yield load (p = 0.003) and max load (p = 0.015) compared to C3H/C3H and C3H/B10 femora. However, there were no significant differences in energy absorbed between sex and genotypes. Generally, mice harboring the C3H alleles had greater phenotypic value than homozygous B10/B10 mice. Thus, these findings demonstrated that B10/B10 femurs were less ductile and had reduced biomechanical properties at the whole-bone level relative to mice harboring C3H allele. Therefore, the short congenic segment of C.B.4.2 mice also governed bone strength differences between genotypes.

**Table 6:** Whole-Bone Mechanical Performance of C.B.4.2 Mice.

C.B. 4.2						
	MALES			FEMALES		
	C3H/C3H (10)	C3H/B10 (17)	B10/B10 (9)	C3H/C3H (4)	C3H/B10 (8)	B10/B10 (6)
**total displacement (mm)**^**A**^	0.54 ± 0.08	0.66 ± 0.05	0.49 ± 0.06	0.62 ± 0.09	0.88 ± 0.15	0.63 ± 0.10
**post-yield displacement (mm)**^**A**^	0.42 ± 0.08	0.53 ± 0.05	0.43 ± 0.05	0.50 ± 0.09	0.77 ± 0.12	0.48 ± 0.10
**stiffness (N/mm)**^**A**^	134 ± 9	123 ± 5	108 ± 6	116 ± 6	125 ± 5	105 ± 10
**yield Load (N)**^**A**^	14.9 ± 0.5	14.3 ± 0.4	12.8 ± 0.6	13.9 ± 1.9	15.0 ± 0.5	12.7 ± 0.9
**max load (N)**^**A**^	19.6 ± 1.0	19.5 ± 0.4	17.3 ± 0.8	19.4 ± 1.2	19.8 ± 0.43	17.8 ± 1.0
energy (N*mm)	4.02 ± 0.63	5.11 ± 0.37	4.31 ± 0.28	4.85 ± 0.12	5.38 ± 0.37	3.86 ± 0.22

Values are means ± SE; N, numbers of mice. Data analyzed by 2 way ANOVA

^A^ genotype effect

#### Tissue Level Properties of C.B.4.2 Mice

Tissue level biomechanical performance is summarized in Table D in S1 File. There were no significant differences in all tissue level properties between sex and genotypes.

#### Additive and Dominance Effects of C.B.4.2 Mice

The additive and dominance effect of the alleles are summarized in Table 7. In males, the locus had a more significant additive effect for OMA and CSA. Conversely, the locus had a larger dominance effect in females resulting in the heterozygote females having larger bone than either homozygote. In both sexes, the locus had a more significant dominance effect for total displacement, PYD, stiffness, yield load and max load. Comparison of additive versus dominance effect using male and female average for each genotype showed a more significant dominance effect for OMA, CSA, total displacement, PYD, stiffness, yield load and max load. In summary, the locus had a significant dominance effect on bone strength.

**Table 7:** Additive and Dominance Effects for Chromosome 4 QTL in C.B.4.2 Mice.

C.B. 4.2					
	**MALES**				
** **	**C3H/C3H (10)**	**C3H/B10 (17)**	**B10/B10 (9)**	**additive effect**	**dominance effect**
**Outer minor axis (mm)**^**A**^^,^^**C**^	1.13 ± 0.02	1.14 ± 0.01	1.16 ± 0.02	0.02	-0.01
**CSA (mm**^**2**^**)**^**A**^^,^^**C**^	1.08 ± 0.04	1.08 ± 0.02	1.14 ± 0.02	0.03	-0.03
**total displacement (mm)**^**A**^	0.54 ± 0.08	0.66 ± 0.05	0.49 ± 0.06	-0.03	0.15
**post-yield displacement (mm)**^**A**^	0.42 ± 0.08	0.53 ± 0.05	0.43 ± 0.05	0.01	0.11
**stiffness (N/mm)**^**A**^	134 ± 9	123 ± 5	108 ± 6	-13.15	2.47
**yield Load (N)**^**A**^	14.9 ± 0.5	14.3 ± 0.4	12.8 ± 0.6	-1.07	0.43
**max load (N)**^**A**^	19.6 ± 1.0	19.5 ± 0.4	17.3 ± 0.8	-1.13	1.11
	**FEMALES**				
	**C3H/C3H (4)**	**C3H/B10 (8)**	**B10/B10 (6)**	**additive effect**	**dominance effect**
**Outer minor axis (mm)**^**A**^^,^^**C**^	1.14 ± 0.01	1.16 ± 0.02	1.08 ± 0.03	-0.03	0.05
**CSA (mm**^**2**^**)**^**A**^^,^^**C**^	1.05 ± 0.03	1.07 ± 0.03	0.95 ± 0.05	-0.05	0.07
**total displacement (mm)**^**A**^	0.62 ± 0.09	0.88 ± 0.15	0.63 ± 0.10	0.01	0.26
**post-yield displacement (mm)**^**A**^	0.50 ± 0.09	0.77 ± 0.12	0.48 ± 0.10	-0.01	0.28
**stiffness (N/mm)**^**A**^	116 ± 6	125 ± 5	105 ± 10	-5.90	14.55
**yield Load (N)**^**A**^	13.9 ± 1.9	15.0 ± 0.5	12.7 ± 0.9	-0.62	1.70
**max load (N)**^**A**^	19.4 ± 1.2	19.8 ± 0.43	17.8 ± 1.0	-0.82	1.19
	**MALES AND FEMALES AVERAGE**				
	**C3H/C3H (14)**	**C3H/B10 (25)**	**B10/B10 (15)**	**additive effect**	**dominance effect**
**Outer minor axis (mm)**^**A**^^,^^**C**^	1.14	1.15	1.12	-0.01	0.02
**CSA (mm**^**2**^**)**^**A**^^,^^**C**^	1.07	1.08	1.05	-0.01	0.02
**total displacement (mm)**^**A**^	0.58	0.77	0.56	-0.01	0.20
**post-yield displacement (mm)**^**A**^	0.46	0.65	0.46	-0.00	0.19
**stiffness (N/mm)**^**A**^	125	124	106	-9.52	8.51
**yield Load (N)**^**A**^	14.4	14.6	12.7	-0.84	1.06
**max load (N)**^**A**^	19.5	19.7	17.5	-0.97	1.15

Values are means ± SE; N, numbers of mice.

^A^ genotype effect

^C^ sex by genotype interaction

Additive effect = ½ [average phenotype (C3H/C3H)–average phenotype (B10/B10)]

Dominance effect = average phenotype (C3H/B10)– ½ [average phenotype (C3H/C3H) + average phenotype (B10/B10)]

## Discussion

Our previous studies using recombinant congenic mice HcB-8 and HcB-23 showed differences in bone mechanical properties while linkage analysis revealed high LOD scores for chromosome 4 QTL effects on bone size, shape and strength [10, 20, 21]. The localization of the chromosome 4 QTL coincides with *Bmd7*, a QTL that has been identified by multiple research groups to have an effect on femoral BMD, geometry and biomechanical performance [13, 38–40]. The results from C.B.4.3 and C.B.4.2 are consistent with the previous findings from HcB-8 and HcB-23. In both C.B.4.3 and C.B.4.2 congenic strains, homozygous B10/B10 had smaller bone and reduced biomechanical performance relative to homozygous C3H/C3H mice, consistent with our prior linkage mapping data. Sex by genotype interaction was also observed in both congenic models. There were few phenotypic differences between C.B.4.3 and C.B.4.2 strains. In C.B.4.3 strain, homozygous B10/B10 mice had smaller CSA than homozygous C3H/C3H mice regardless of sex. In the C.B.4.2 strain, homozygous B10/B10 female mice had smaller CSA than the other genotypes, while homozygous B10/B10 male mice tended toward larger CSA. The effect of the short congenic segment harboring homozygous B10/B10 alleles therefore appeared to be opposite depending on the sex. The short congenic segment of C.B.4.2 appeared to have greater structural effect between sexes only on mice homozygous for B10 alleles. There were more significant differences between genotypes in whole-bone biomechanical properties in C.B.4.2 mice compared to C.B.4.3. Sex by genotype interaction was observed for both stiffness and max load in C.B.4.3 mice. In C.B.4.2 strain, homozygous C3H/C3H mice had higher stiffness, yield load and max load than homozygous B10/B10 mice for both sexes. In general, homozygous B10/B10 mice had reduced biomechanical properties relative to homozygous C3H/C3H mice for both congenic strains, in keeping with our earlier findings in the HcB-8 x HcB-23 intercross. The lack of tissue level phenotypes differences suggests that the primary effect of the QTL is on growth, as we previously inferred. Collectively, these findings confirmed that the chromosome 4 QTL regulates differences in bone geometry and biomechanical performance.

Neither C.B.4.3 nor C.B.4.2 mice exhibited the complete set of phenotypic differences found in the intercross, with C.B.4.3 displaying most of the femoral size and shape phenotypes and C.B.4.2 mice exhibiting more of the biomechanical performance phenotypes observed in the original intercross. These findings support the interpretation that multiple allelic differences within distal chromosome 4 influence bone properties, as previously proposed [11]. In the course of constructing these strains, genotyping for additional markers revealed that HcB-8 harbors a larger, but non-contiguous contribution of the B10 genome than we appreciated at the time of our earlier experiments. A congenic mouse strain B6.C3H-4T (4T) that harbors 70 Mb of C3H sequence on a B6 background, and includes the congenic segments studied here, showed higher femoral and vertebral volumetric bone mineral density compared to B6 controls [11, 38]. Previous *in vivo* ulna loading study using 4T mice showed differences in skeletal adaptation to mechanical loading compared to B6 control mice [12]. The mid-distal region of mouse chromosome 4 shares sequence homology with human chromosome 1 p36 (Chr 1p36) across a region of ~24 Mb and contains multiple QTL regions for which distinct skeletal phenotypes have been reported [8, 11]. In humans, early Genome Wide Associated Studies (GWAS) investigations reported human Chr 1p36 as an important region for bone mineral density regulation at the hip, lumbar spine, femoral neck and wrist [41–44]. Due to these consistent findings, many efforts have been made in search for potential bone regulatory genes within the Chr1p36 region [45–49]. Despite the effort, little progress has been made in finding a consensus on a gene or set of genes important to bone physiology. Few of the confounding variables include: 1) human genome heterogeneity, 2) human sample sizes, 3) epigenetic factors, and 4) sex and age differences. These factors also pose limitations in characterizing gene to gene and gene to environment interactions. The significant difference in biomechanical performance of C.B.4.2 mice supports our earlier inference that *Ece1*, encoding endothelin converting enzyme 1, can potentially contribute to the effect of the QTL [10]. *Ece1* lies within both the C.B.4.3 and the much smaller C.B.4.2 congenic segments, and thus is strengthened as a candidate gene by the findings reported here.

Eighteen chromosome 4 congenic strains were derived from the distal portion of chromosome 4 in 4T mice [11]. In 4T-derived congenic strains, introduction of C3H sequence to the B6 background had phenotypic effects on BMD, bone size and volume relative to B6 controls. From this study, 10 QTL regions in the mid distal portion of chromosome 4 were identified. Within these regions, 13 candidate genes, that may have functional implications on bone mineralization, size and architectural regulation, were inferred from mouse genome databases [11]. Interaction with other genes within the congenic segment also cannot be rule out. Currently, there are no functional studies on the effect of these candidate genes on bone phenotypes. Our lab has constructed both long (~20 Mb) and short congenic segment (~3 Mb) of the chromosome 4 QTL that contain one of the candidate genes, *Ece1* [10]. In addition, we have also done mechanical studies and mid-diaphyseal geometry analysis of the bone. There are several differences between the 4T mice studies versus our congenic studies. Our congenic models used B10 alleles as the donor strain on a C3H background while the 4T mice used C3H alleles as the donor strain on a B6 background. The 4T mice harbor ~40 Mb C3H segment while our C.B.4.3 and C.B.4.2 congenic mice harbor ~20 Mb and ~3 Mb B10 derived chromosome segment respectively [12, 50]. In the 4T model, mice ulnae were used while we used femora for our mechanical loading study. The 4T mice had larger total area and cortical area in comparison to the B6 control group [12]. Similar findings were observed in our C.B.4.3 congenic strains, in which mice homozygous for the C3H genotype have larger CSA relative to mice heterozygous for C3H/B10 genotype and homozygous for B10 genotype. In our C.B.4.2 congenic group, both homozygous C3H females and heterozygous C3H/B10 female mice had significantly larger CSA than homozygous B10/B10 females. On average, 4T mice ulnae were slightly less compliant then B6 mice, but the difference was not significant. In both C.B.4.3 and C.B.4.2 mice, the femora of mice homozygous for B10 genotypes were less ductile than the femora of homozygous C3H mice. Both the 4T and our congenic models showed that chromosome 4 QTL harbors bone relevant genes that play important roles in the regulation of bone biomechanical performance. It has been reported that several QTLs have been mapped for femoral width with highly significant linkage identified on chromosome 4 [51].

The work presented here has several strengths. The use of littermate controls controlled for the potential presence of unidentified donor segments within the genome. In doing so, the effect of genetic drift that may occur during the development of the congenic strains was also eliminated. By studying both femora of each mouse, we obtained better phenotype estimates than would be possible using only a single femur from each animal. By performing comprehensive phenotyping of biomechanical performance and bone structure, we obtained a more detailed characterization than would be achievable by either densitometry or micro-CT alone. Finally, by studying both males and females, we were able to recognize genotype x sex interactions.

There are also important limitations to the work presented here. Since DXA calculates BMD using area, the method does not provide measurement of the volumetric BMD and therefore is biased in favor of larger bones. This limitation may contribute to our failure to find any significant differences in BMD in these experiments. Mechanical testing is different from clinical fractures but it provides a better assessment on bone performance than BMD measurement alone. Biomechanical test are less reproducible than densitometry, in part due to reliance on consistent specimen positioning for the 3 point bending test [52]. A similar limitation also applies to the cross sectional geometry analysis. Both the long and short congenic segments each contain several million bases of DNA, so it is impossible to attribute the phenotypic differences to a specific sequence variant. We have also only analyzed F2 progenies at 17 weeks; therefore we did not address developmental phenotypes during growth or after maturity and the phenotypes of aging bone. Since gonadectomy was not performed, we were unable to address whether differences in the bone phenotypes between males and females are mediated by hormonal regulation.

This study confirms our earlier findings from the recombinant congenic mouse, HcB-8 and HcB-23 studies and justifies our ongoing functional studies of *Ece1*. Our congenic models also showed consistent results with the previously reported phenotypes of 4T mice, despite the converse donor/background combination. Nevertheless, until an *in vivo* demonstration of the effect of *Ece1* on important bone phenotypes is provided, it must be considered as a candidate gene within the various chromosome 4 congenic segments, rather than as the primary gene underlying the major bone QTL in the region.

## Supporting Information

S1 File In vivo measurements and tissue level mechanical performance of the congenic mouse strains. C.B. 4.3 *In vivo* measurements (Table A in S1 File), Tissue Level Mechanical Performance of C.B.4.3 Mice (Table B in S1 File), C.B. 4.2 *In vivo* measurements (Table C in S1 File), and Tissue Level Mechanical Performance of C.B.4.2 Mice (Table D in S1 File).
(DOCX)
