# Supplementary material for: Congenic Strains Confirm the Pleiotropic Effect of Chromosome 4 QTL on Mouse Femoral Geometry and Biomechanical Performance
Source: PLoS One. 2016 Feb 5;11(2):e0148571. doi: 10.1371/journal.pone.0148571 (PMC4743951; doi:10.1371/journal.pone.0148571)
Supplement: S1 File — C.B. 4.3 In vivo measurements (Table A in S1 File), Tissue Level Mechanical Performance of C.B.4.3 Mice (Table B in S1 File), C.B. 4.2 In vivo measurements (Table C in S1 File), and Tissue Level Mechanical Performance of C.B.4.2 Mice (Table D in S1 File). (DOCX) [file pone.0148571.s001.docx]

**Supporting Information**

**S1. File. In vivo measurements and tissue level mechanical performance of the congenic mouse strains**

**Table A. C.B. 4.3 *In vivo* measurements**

| **C.B. 4.3** | | | | | | |
| --- | --- | --- | --- | --- | --- | --- |
|  | **MALES** | | | **FEMALES** | | |
|  | **C3H/C3H (9)** | **C3H/B10 (8)** | **B10/B10 (10)** | **C3H/C3H (8)** | **C3H/B10 (9)** | **B10/B10 (8)** |
| **Body weight (g)^B^** | 24.6 ± 0.6 | 23.0 ± 0.5 | 23.3 ± 0.7 | 21.2 ± 0.4 | 21.0 ± 0.5 | 22.4 ± 0.4 |
| **Rostro-anal length (cm)** | 9.3 ± 0.1 | 9.1 ± 0.1 | 9.4 ± 0.1 | 9.1 ± 0.1 | 9.2 ± 0.1 | 9.2 ± 0.1 |
| **femur length (mm)** | 14.62 ± 0.12 | 14.51 ± 0.15 | 14.23 ± 0.19 | 14.84 ± 0.07 | 14.71 ± 0.13 | 14.95 ± 0.04 |
| **femur BMD (mg/cm^2^)** | 60 ± 1 | 65 ± 3 | 64 ± 3 | 63 ± 3 | 70 ± 4 | 62 ± 3 |

Values are means ± SE; N, numbers of mice for measurement of body weight, body length and femur length. Data analyzed by 2 way ANOVA

^A^ genotype effect

^B^ sex effect

^C^ sex by genotype interaction

**Table B. Tissue Level Mechanical Performance of C.B.4.3 Mice**

| **C.B. 4.3** | | | | | | | |
| --- | --- | --- | --- | --- | --- | --- | --- |
|  | **MALES** | | | **FEMALES** | | |  |
|  | **C3H/C3H (9)** | **C3H/B10 (8)** | **B10/B10 (10)** | **C3H/C3H (8)** | **C3H/B10 (9)** | **B10/B10 (8)** |  |
| **Total Strain (unitless)** | 0.050 ± 0.003 | 0.040 ± 0.009 | 0.055 ± 0.007 | 0.070 ± 0.009 | 0.058 ± 0.007 | 0.044 ± 0.007 |  |
| **Yield Strain (unitless)** | 0.020 ± 0.005 | 0.030 ± 0.006 | 0.038 ± 0.010 | 0.05 ± 0.008 | 0.038 ± 0.006 | 0.028 ± 0.007 |  |
| **PY Strain (unitless)^B^** | 0.030 ± 0.004 | 0.030 ± 0.011 | 0.026 ± 0.002 | 0.020 ± 0.002 | 0.020 ± 0.002 | 0.016 ± 0.002 |  |
| **Young's Modulus (MPa)** | 5716 ± 1221 | 5695 ± 1266 | 6349 ± 1443 | 5501 ± 1526 | 5847 ± 1116 | 7355 ± 768 |  |
| **Yield Stress (MPa)** | 100 ± 24 | 105 ± 24 | 107 ± 22 | 98 ± 23 | 109 ± 20 | 144 ± 10 |  |
| **Max Stress (MPa)** | 131 ± 29 | 125 ± 28 | 145 ± 32 | 118 ± 30 | 128 ± 24 | 148 ± 15 |  |
| **Toughness (MPa)** | 3.87 ± 0.91 | 3.00 ± 0.60 | 3.91 ± 0.71 | 2.72 ± 0.55 | 3.30 ± 0.50 | 3.79 ± 0.54 |  |
| **Failure Stress (MPa)** | 129 ± 29 | 124 ± 27 | 141 ± 30 | 113 ± 29 | 126 ± 24 | 146 ± 15 |  |

Values are means ± SE; N, numbers of mice. Data analyzed by 2 way ANOVA

^A^ genotype effect

^B^ sex effect

^C^ sex by genotype interaction

**Table C. C.B. 4.2 *In vivo* measurements**

| **C.B. 4.2** | | | | | | |
| --- | --- | --- | --- | --- | --- | --- |
|  | **MALES** | | | **FEMALES** | | |
|  | **C3H/C3H (10)** | **C3H/B10 (17)** | **B10/B10 (9)** | **C3H/C3H (4)** | **C3H/B10 (8)** | **B10/B10 (6)** |
| **Body weight (g)^B^** | 23.1 ± 0.9 | 23.8 ± 0.4 | 25.2 ± 0.6 | 20.3 ± 0.3 | 20.4 ± 0.3 | 19.6 ± 0.6 |
| **Rostro-anal length (cm)** | 9.1 ± 0.1 | 9.3 ± 0.1 | 9.4 ± 0.1 | 9.1 ± 0.1 | 9.1 ± 0.1 | 9.0 ± 0.1 |
| **femur length (mm)** | 13.97 ± 0.25 | 14.34 ± 0.11 | 14.68 ± 0.08 | 14.40 ± 0.21 | 14.58 ± 0.14 | 14.32 ± 0.17 |
| **femur BMD (mg/cm^2^)** | 61 ± 2 | 68 ± 2 | 66 ± 3 | 64 ± 1 | 69 ± 1 | 67 ± 1 |

Values are means ± SE; N, numbers of mice for measurement of body weight, body length and femur length. Data analyzed by 2 way ANOVA

^A^ genotype effect

^B^ sex effect

^C^ sex by genotype interaction

**Table D. Tissue Level Mechanical Performance of C.B.4.2 Mice**

| **C.B. 4.2** | | | | | | |
| --- | --- | --- | --- | --- | --- | --- |
|  | **MALES** | | | **FEMALES** | | |
|  | **C3H/C3H (10)** | **C3H/B10 (17)** | **B10/B10 (9)** | **C3H/C3H (4)** | **C3H/B10 (8)** | **B10/B10 (6)** |
| **Total Strain (unitless)** | 0.032 ± 0.005 | 0.036 ± 0.005 | 0.033 ± 0.003 | 0.025 ± 0.006 | 0.037 ± 0.006 | 0.030 ± 0.005 |
| **Yield Strain (unitless)** | 0.015 ± 0.004 | 0.017 ± 0.004 | 0.013 ± 0.001 | 0.012 ± 0.003 | 0.019 ± 0.005 | 0.022 ± 0.008 |
| **PY Strain (unitless)** | 0.019 ± 0.003 | 0.029 ± 0.003 | 0.023 ± 0.002 | 0.023 ± 0.007 | 0.028 ± 0.007 | 0.024 ± 0.004 |
| **Young's Modulus (MPa)** | 7496 ± 1194 | 5988 ± 745 | 6145 ± 929 | 5889 ± 2633 | 6913 ± 1388 | 6174 ± 1164 |
| **Yield Stress (MPa)** | 98 ± 15 | 87 ± 10 | 88 ± 14 | 86 ± 26 | 92 ± 21 | 83 ± 14 |
| **Max Stress (MPa)** | 147 ± 15 | 140 ± 11 | 128 ± 19 | 107 ± 41 | 138 ± 28 | 152 ± 30 |
| **Toughness (MPa)** | 6.51 ± 1.03 | 7.75 ± 0.95 | 6.47 ± 1.11 | 5.66 ± 2.44 | 8.02 ± 2.03 | 7.30 ± 1.99 |
| **Failure Stress (MPa)** | 145 ± 15 | 135 ± 11 | 120 ± 22 | 103 ± 40 | 129 ± 26 | 135 ± 26 |

Values are means ± SE; N, numbers of mice. Data analyzed by 2 way ANOVA

^A^ genotype effect

^B^ sex effect

^C^ sex by genotype interaction
